# Metabolomics Analysis of Seminal Plasma in Infertile Males with Kidney-Yang Deficiency: A Preliminary Study

**DOI:** 10.1155/2015/892930

**Published:** 2015-04-07

**Authors:** Xiang Chen, Chao Hu, Jican Dai, Lei Chen

**Affiliations:** ^1^Department of Urology, Ren Ji Hospital, School of Medicine, Shanghai Jiao Tong University, Shanghai 200001, China; ^2^Department of Urology, Zhongshan Hospital, Fudan University, Shanghai 20032, China; ^3^Department of Urology, Longhua Hospital, Shanghai University of Traditional Chinese Medicine, Shanghai 200032, China

## Abstract

Traditional Chinese medicine (TCM) is an important treatment for male infertility, and its application to therapy is dependent on differentiation of TCM syndromes. This study aims to investigate the changes in metabolites and metabolic pathways in infertile males with Kidney-Yang Deficiency syndrome (KYDS) via metabolomics approaches. Seminal plasma samples were collected from 18 infertile males with KYDS and 18 fertile males. Liquid chromatography and mass spectrometry were used to characterize metabolomics profiles. Principal component analysis (PCA), partial least squares-discriminate analysis (PLS-DA), and pathway analysis were used for pattern recognition and metabolite identification. PCA and PLS-DA results differentiated the two groups of patients. Forty-one discriminating metabolites (18 in positive mode and 23 in negative mode) were identified. Seven metabolites were related to five potential metabolic pathways associated with biosynthesis and metabolism of aromatic amino acids, tricarboxylic acid cycle, and sphingolipid metabolism. The changes in metabolic pathways may play an important role in the origin of KYDS-associated male infertility. Metabolomics analysis of seminal plasma may be used to differentiate TCM syndromes of infertile males, but further research must be conducted.

## 1. Introduction

Male infertility inflicts 15%–20% of couples, and 30% of infertility cases are defined as “idiopathic infertility,” which has no discernible cause [1]. Male infertility with apparent causes, such as obstructive azoospermia and hypogonadotropic hypogonadism, can be cured through surgery and endocrinotherapy. In the absence of evident causality, empirical pharmacologic therapies with gonadotropins, antiestrogens, and antioxidants are administered; nevertheless, the effects are uncertain [1]. The invention of assisted reproductive technology (ART), an effective therapy for infertility, provides affected couples with the possibility of conception. Although ART can induce pregnancy in many cases, regardless of infertility causes, it is more complicated and painful than medication. Moreover, as ART may not be covered in insurance claims, patients cannot afford its expensive cost. Hence, alternative medicines, particularly traditional Chinese medicine (TCM) and acupuncture, which are popular in Asia, have been widely used for treatment of male infertility [2, 3].

Metabolomics is the study of small low-molecular-weight metabolites in complex biological samples, aiming to characterize and quantify all the small molecules in such samples [4]. Though in its infancy, metabolomics, as well as genomics and proteomics [5], has been used to identify biomarkers of male infertility and presents a potential application for semen analysis and gamete and embryo selection [6]. Metabolome represents the functional state of an individual at a particular time point and is similar to TCM theory; this theory emphasizes holism, considers diseases from various dynamic functional aspects, and utilizes individualized herbal formula based on TCM syndrome for treatments [7]. Given the advantages of metabolomics, many studies used this method to explore the mechanisms of TCM syndromes [8] and the effect of Chinese herbal medicines [7]. Nevertheless, metabolomics has been rarely employed to elucidate the TCM syndromes of male infertility.

In TCM theory, male infertility is closely related to disorders of the spleen, liver, and especially kidney [3]. A study on 800 Chinese infertile males [9] revealed that their TCM syndromes appeared concurrently, not separately, in most cases. In this study group, 74.9% of the patients suffered from Kidney-Yang Deficiency syndrome (KYDS), showing that KYDS is the predominant TCM syndrome in infertile males.

The fecundity of males mostly depends on semen quality, and metabolites in seminal plasma reflect the metabolic state of spermatozoa. Previous studies showed that metabolomics analysis can be used to differentiate males with low sperm concentration [10] or asthenozoospermia [11] from normal. A variety of techniques such as mass spectrometry, nuclear magnetic resonance spectroscopy, and Fourier transform infrared spectroscopy are available in metabolomics [12] at present. These are useful tools for screening of metabolites and biomarkers, and among them liquid chromatography-mass spectrometry (LC-MS) had already been used in metabolomics studies of seminal plasma [13]. In order to approach the essence of KYDS in infertile males and to find a modernized, objective method for TCM syndrome diagnosis in male infertility, seminal plasma samples from infertile males with KYDS and fertile males were analyzed using the technique of LC-MS.

## 2. Materials and Methods

### 2.1. Participants and Selection Criteria

This study was approved by the Local Ethics Committee of Ren Ji Hospital. Semen samples were collected from infertile males with KYDS in the Male Infertility Clinic of Ren Ji Hospital, Shanghai. The latest semen analysis results of the patients were recorded. Infertility was defined as the absence of conception after 12 months of regular, unprotected intercourse [1]. Infertile couples with female factors and males who failed to achieve intravaginal ejaculation were excluded from our study. The diagnostic criteria for KYDS were as follows [9]. Primary manifestations included (1) abnormal semen quality, such as oligozoospermia (sperm concentration < 20 × 10^6^/mL) and asthenozoospermia (less than 50% motile spermatozoa or less than 25% spermatozoa with progressive motility); (2) sexual dysfunction, such as loss of libido, erectile dysfunction, and premature ejaculation; and (3) weak ejaculation or orgasm disorder. Secondary manifestations included (1) pain or coldness in the back or knees; (2) fatigue, lethargy, or weakness; (3) increased urine volume or frequency of micturition; (4) thready or deep pulse; and (5) pale tongue with a thin and white coating. Patients with two primary and two secondary manifestations were enrolled in the study, and diagnosis was provided by the same physician.

Semen samples of fertile males were collected from Shanghai Human Sperm Bank. The criteria of fertile males were defined as follows: (1) sperm donors of Chinese nationality, (2) married with age between 20 and 40 years old, (3) spouse has undergone pregnancy or delivery, (4) screening of medical history, physical examination, and laboratory tests on the basis of the “Recommendations for Gamete and Embryo Donation” [14], and (5) semen analysis results of sperm concentration ≥ 60 × 10^6^/mL and other seminal parameters within the WHO 1999 normal reference values [15].

### 2.2. LC-MS Analysis

Seminal plasma samples were purified through centrifugation of semen (3000 rpm, 10 min, and 25°C). Supernatant (seminal plasma) was collected and stored at −20°C until further analysis. Prior to LC-MS analysis, 300 *μ*L of methanol was added to 100 *μ*L of seminal plasma and then vortex mixed for 1 min. The sample mixture was then centrifuged at 12000 rpm for 10 min at 4°C to remove proteins. To ensure reproducibility, we prepared a quality control sample by pooling equal volumes (10 *μ*L) of each seminal plasma sample. The pooled sample was also subjected to similar protein precipitation procedure. All the samples were randomly run through analytical batch. In addition, six aliquots of seminal plasma samples from one man were treated with the same process to assess reproducibility.

Sample fingerprinting was performed on an Agilent 1290 LC system with an autosampler and a binary pump coupled to Agilent Q-TOF 6530 (Agilent, Palo Alto, CA, USA). Chromatographic separation was performed on an Agilent C_18_ column (1.8 *μ*m, 2.1 mm × 100 mm) with the column temperature maintained at 40°C. The flow rate was 0.4 mL/min, and the mobile phase consisted of ultrapure water with 0.1% (v/v) formic acid (A) and acetonitrile with 0.1% (v/v) formic acid (B). The gradient program is shown in Additional File 1 in Supplementary Material available online at http://dx.doi.org/10.1155/2015/892930. The sample injection volume was 4 *μ*L.

The mass spectrometer was operated in positive and negative ion modes. Nitrogen was used as nebulizer gas with a flow rate of 8 L/min, scan time of 0.03 s, interscan time of 0.02 s, and scan range of 50–1000 *m*/*z*.

Parameters for positive ion mode were as follows: capillary voltage of 4 kV, sampling cone voltage of 35 kV, source temperature of 100°C, desolvation temperature of 350°C, cone gas flow rate of 50 L/h, desolvation gas flow rate of 600 L/h, and extraction cone voltage of 4 V.

Parameters for negative ion mode were as follows: capillary voltage of 3.5 kV, sampling cone voltage of 50 kV, source temperature of 100°C, desolvation temperature of 300°C, cone gas flow rate of 50 L/h, desolvation gas flow rate of 700 L/h, and extraction cone voltage of 4 V.

### 2.3. Data Processing and Analysis

The open-source XCMS software (http://metlin.scripps.edu/download/) was used for preprocessing of LC-MS data. Retention time, mass-to-charge ratio (*m*/*z*), and intensity for each sample were reformed into an Excel matrix. The matrix was then imported into SIMCA-P software (Version 11.5, Umetrics AB, Sweden) for multivariate statistical analysis. Principal component analysis (PCA) and partial least squares-discriminate analysis (PLS-DA) were employed to identify biochemical patterns. The values of variable importance in the projection (VIP) in PLS-DA model combined with the *P* value of Student's *t*-test were used to determine important metabolites. Univariate analysis was performed using SPSS 11.5 (SPSS Inc., Chicago, IL, USA), and *P* values lower than 0.05 were considered significant. The exact molecular mass and *m*/*z* were used to identify the characteristic metabolites in the Human Metabolome Database (http://www.hmdb.ca/) and Metabolites and Tandem MS Database (http://metlin.scripps.edu). The identified metabolites were then confirmed by comparing their accurate mass, retention time, and fragments with the commercial standards. Potential biomarkers were subjected to pathway analysis with MetPA software (http://metpa.metabolomics.ca./MetPA/faces/Home.jsp) based on the KEGG Pathway Database (http://www.genome.jp/kegg/pathway.html) to identify related metabolic pathways.

## 3. Results

### 3.1. Semen Analysis of Infertile Males with KYDS

A total of 18 patients underwent semen analysis. All were married. The mean age of infertile males with KYDS was 31.4 (range from 23 to 37 years). Abnormalities observed in semen analysis of these individuals are shown in Table 1. Asthenozoospermia and teratozoospermia were the most prevalent spermatozoan abnormalities. Delayed liquefaction, low pH, and low semen volume were also common in our patients. Although these conditions are not the major cause of infertility, they reflect some changes in the reproductive system of infertile men with KYDS. Fifteen patients presented more than one abnormality in semen analysis, and one patient had a normal semen analysis result.

### 3.2. PCA and PLS-DA of Metabolomics Profiles

**Figure 1:**
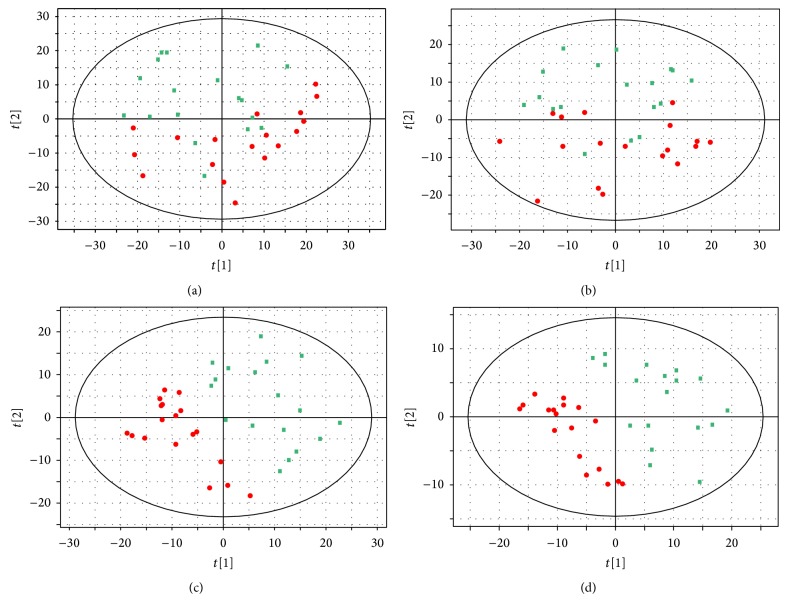
Score plots of PCA and PLS-DA. Multivariate analysis of metabolic profiles of seminal plasma samples from infertile males with KYDS and fertile males. (a) PCA analysis in positive mode, (b) PCA analysis in negative mode, (c) PLS-DA analysis in positive mode, and (d) PLS-DA analysis in negative mode. Each dot represents data from a seminal plasma sample. Green represents the fertile males, and red represents infertile males with KYDS.

PCA, an unsupervised pattern recognition method, was used to determine the presence of inherent similarities in spectral profiles. Each scatter represented a patient's seminal sample. Although PCA results partially overlapped in positive and negative ion modes (Figures 1(a) and 1(b), resp.), the distribution of the two groups differed. Variances of 38.3% and 36.1% were explained by the first two principal components of PCA analysis in positive and negative ion modes, respectively. To identify discriminating metabolites and differentiate the two groups, we used the corresponding PLS-DA analysis.

The PLS-DA loading plot showed that the scatters of the two groups were completely separated (Figures 1(c) and 1(d)), which could be attributed to differential metabolites. The performance characteristics of this multivariate model for positive ion mode were as follows: *R* ^2^ (*X*) = 0.428, *R* ^2^ (*Y*) = 0.874, and *Q* ^2^ = 0.579. Similarly, the performance characteristics of the model for negative ion mode were as follows: *R* ^2^ (*X*) = 0.234, *R* ^2^ (*Y*) = 0.799, and *Q* ^2^ = 0.425. These results demonstrated the existence of different seminal plasma biological signatures between infertile males with KYDS and fertile males.

### 3.3. Metabolite Identification and Pathway Analysis

**Figure 2:**
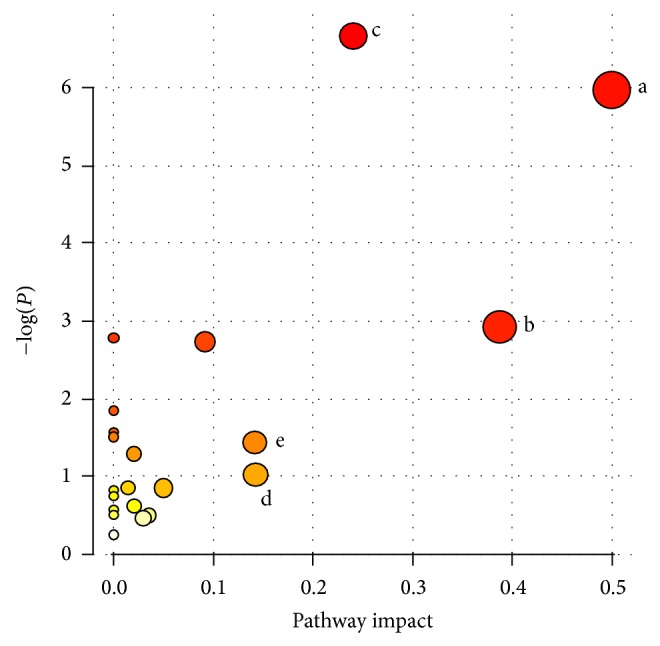
Summary of pathway analysis with MetPA. (a) Phenylalanine, tyrosine, and tryptophan biosynthesis, pathway impact: 0.50, (b) citric acid cycle, pathway impact: 0.39, (c) phenylalanine metabolism, pathway impact: 0.24, (d) sphingolipid metabolism, pathway impact: 0.14, and (e) tyrosine metabolism, pathway impact: 0.14. The node color is based on its *P* value and the node radius is based on their pathway impact values.

The typical LC-MS total ion chromatograms are shown in Additional File 2. A total of 41 discriminating metabolites (VIP > 1.0, *P* < 0.05), including 18 in positive mode and 23 in negative mode (Tables 2 and 3), were identified in seminal plasma. Most metabolites decreased in infertile males with KYDS. The possible pathways related to the conditions under study were identified with MetPA, a free tool based on the high-quality KEGG metabolic pathways database [17]. The pathway impact value was calculated from pathway topology analysis. Pathways with values higher than 0.1 were screened as potential target pathways and might be used to differentiate infertile males with KYDS from fertile males. The five potential pathways were phenylalanine metabolism, phenylalanine, tyrosine, and tryptophan biosynthesis, glyoxylate and dicarboxylate metabolism, tyrosine metabolism, and sphingolipid metabolism (Figure 2).

## 4. Discussion

TCM syndrome is based on the understanding of the regularity of disease occurrence and development as well as a certain stage of a comprehensive response of patients with a certain condition [18]. Differentiation of TCM syndromes is based on the human body's overall features, including pulse manifestation, tongue, tongue-coating changes, and symptoms; hence, it is more subjective and different from modern medicine. As therapy with customized herbal formula mainly depends on TCM syndrome differentiation, translation of this ancient system into modern science is important.

Investigation of the intricate mechanism of TCM syndrome is difficult until the emergence of “Omics.” This field of study is widely used to investigate the essence of TCM syndrome [19], for its method and design resemble TCM theory which focuses on the integrity of disease and overall health state. Among all “Omics,” metabolomics is particularly important because it reflects the most downstream metabolite information, which is the direct response to pathophysiological changes caused by diseases.

Specific diagnostic criteria for TCM syndromes have been developed since the 1980s, but most of them are based on symptoms and subjective estimates in pulse conditions [20]. For some infertile males without symptoms or manifestations, the changes used to differentiate TCM syndromes may be absent [21]. As a result, they could not be easily treated with TCM formulas. Therefore, an objective, sensitive, and exact experimental model for differentiation of TCM syndromes must be developed to manage this group of patients. Metabolomics could be utilized as a microcosmic tool for diagnosis of TCM syndromes.

Our study revealed different metabolites and metabolic pathways associated with KYDS via LC-Q-TOF-MS, a sensitive metabolomics technique. The PCA and PLS-DA plots differed between seminal plasma of fertile males and infertile males with KYDS (Figure 1), which indicates the presence of different metabolites. Gilany et al. [11] used spectroscopy combined with chemometrics to assess differences in the metabolome of seminal plasma of patients with asthenozoospermia; the controls used were men with normal semen quality according to the WHO 2010 criteria [16]. The results showed that the two groups differed; thus, they developed a prediction model with a validity of 83%. Courant et al. [10] also used LC-MS to generate metabolomics fingerprints from plasma samples collected from young Danish men presenting low (>0–20 × 10^6^/mL), intermediate (45–75 × 10^6^/mL), or high (>100 × 10^6^/mL) sperm concentrations. The serum metabolic profiles significantly differed among the three groups. These two studies indicate that metabolic differences affect sperm concentration and motility. In the present study, we presented a similar conclusion, but infertile males with KYDS were not all oligozoospermic or asthenozoospermic (Table 1). Oligozoospermia or asthenozoospermia could partially explain male infertility with KYDS. Furthermore, infertility in males with KYDS may be attributed to DNA fragmentation, failure of acrosomal reaction, and so on [22].

Courant et al. [10] found 16 metabolites that contributed to differentiation of sperm concentration. In the present research, 41 metabolites were related to infertile males with KYDS (Tables 2 and 3). Both studies found tyrosine, aconitic acid, and pyroglutamic acid as differential metabolites. The different specimen source and metabolite data banks used in the two studies possibly contributed to the discrepancy in results. In our study, we obtained only one patient with oligozoospermia and six with asthenozoospermia in the 18 infertile men (Table 1). The composition of patients group may also contribute to the difference in metabolites. These results accord with the former discovery that asthenozoospermia appeared more often in infertile males with KYDS than oligozoospermia [23], suggesting that asthenozoospermia is more prevalent and important than oligozoospermia in infertile males with KYDS.

In this study, 21 metabolic pathways related to 41 discriminating metabolites were found in seminal plasma, which is a complex biological sample. The changes in intensity between the two groups were less than twofold for each metabolite (Tables 2 and 3), and most of the pathways identified by MetPA had low impact values. A decreasing trend was observed in most metabolite levels in KYDS samples compared with that in the controls; this decrease resembles the meaning of “deficiency” in KYDS. However, this trend did not coincide with the urine or serum metabolomics profiles in an animal model with Kidney-Yang Deficiency induced by hydrocortisone [24, 25]. This difference showed the complexity of KYDS. KYDS subtypes may exist since not all Kidney-Yang Deficiency patients were infertile.

The following seven metabolites were related to the five potential metabolic pathways: 2-phenylacetamide, phenylpyruvic acid, tyrosine, homovanillic acid, citric acid, aconitic acid, and sphinganine (Figure 2). 2-Phenylacetamide, phenylpyruvic acid, and tyrosine were associated with the biosynthesis and metabolism of aromatic amino acids. These three metabolites decreased in infertile males with KYDS compared with those in fertile males. Research on mice with KYDS induced by purine [26] showed a decrease in the adrenocorticotropic hormone compared with the normal controls. These effects could be reversed by using tonics for KYDS. The use of* Jin Kui Shen Qi Wan*, a tonic for KYDS, could enhance the incorporation of ^3^H-tyrosine in pituicytes and may reverse the effect of KYDS by enhancing protein biosynthesis [26]. The decrease in 2-phenylacetamide, phenylpyruvic acid, and tyrosine indicated the decrease in protein biosynthesis in spermatozoa, which contributed to the generation of KYDS. Citric and aconitic acids, which are associated with the citric acid cycle, presented a decreasing trend in infertile males with KYDS. The citric acid cycle or tricarboxylic acid cycle, which occurs in the mitochondria, is the key point of aerobic respiration. This metabolic pathway is closely related to the motion of spermatozoa. A previous proteomic study [27] found that the use of KYDS tonics reversed the inhibited citric acid cycle and beta-oxidation of fatty acids caused by KYDS; as a result, energy metabolism was alleviated via increased output of ATP in hepatocytes. These findings indicate that the impaired energy metabolism in semen may be attributed to KYDS. Moreover, the levels of sphinganine (dihydrosphingosine), a precursor of sphingosine-1-phosphate, decreased. Sphingosine-1-phosphate, a significant molecule in testis, can inhibit male germ cell apoptosis in the testis [28] and protect testicular germ cells against radiation-induced cell death [29]. And this molecule is also involved in acrosomal exocytosis [30]. The decreased precursor may also lead to teratozoospermia and low vitality, which are common conditions observed in our patients (Table 1), or impaired acrosome reaction.

One of the limitations of our study was insufficiency in seminal samples. Only 18 samples were used for each group, which is a small number for such a complicated disease. A study with larger sample scale should be conducted to establish a metabolomics diagnostic model. As semen quality is fluctuating, a single semen analysis is not enough to diagnose oligoasthenoteratozoospermia as usually two to three semen samples are needed [16]. This principle may be applied in metabolomics profiles, which indicates that the metabolomics profiles of seminal plasma may require repetitive tests. To verify the upstream changes in metabolites, further studies must be conducted using proteomics and transcriptomics.

The opposite side of KYDS, namely, Kidney-Yin Deficiency syndrome, was not investigated in our study because of financial limitations. Studies must be performed to investigate whether Kidney-Yin Deficiency exhibits inverse metabolomics changes. A follow-up metabolomics analysis of patients at post-TCM therapy must also be conducted.

## 5. Conclusions

We compared the seminal plasma metabolomics profiles of normal fertile males and infertile males with KYDS by using LC-MS technique to determine their differences in terms of metabolites and metabolic pathways. To our knowledge, this study is the first to investigate the mechanisms of TCM syndromes on the basis of seminal plasma metabolomics analysis. Infertile males with KYDS were differentiated from fertile males via multivariate statistical analysis. Biosynthesis and metabolism of aromatic amino acids, citric acid cycle, and sphingolipid metabolism contributed to the differentiation. KYDS may affect spermatozoa via these pathways. Evaluation of the metabolomics profiles in seminal plasma may be a powerful tool for investigating the associated mechanisms and microscomically differentiating the TCM syndromes of male infertility.

## Supplementary Material

Additional file 1 was table of gradient elution program of mobile phase. Additional file 2 were typical total ion chromatograms of seminal plasma samples. (a) showed a typical total ion chromatogram from sample of fertile males in positive ion mode, (b) showed a sample of fertile males in negative ion mode, (c) showed a sample of infertile males with KYDS in positive ion mode, and (d) showed a sample of infertile males with KYDS in negative ion mode.

## Figures and Tables

**Table 1:** Abnormity in semen analysis of infertile males with KYDS.

Abnormity in semen analysis	Number of patients
Oligozoospermia	1
Asthenozoospermia	6
Teratozoospermia	7
Leukospermia	1
Low vitality	5
Low semen volume	8
Delayed liquefaction	7
Low semen pH	13

^∗^The WHO 1999 normal reference values [15] were used for the assessment of semen analysis, except for the assessment of teratozoospermia and vitality, which are based on the WHO 2010 criteria [16].

**Table 2:** Identified discriminating metabolites in positive mode.

VIP	*m*/*z*	Retention time (min)	Name	*t*-test	Fold change^*^
1.35	141.1077	1.111	2-Keto-6-aminocaproic acid	0.024	0.688
1.75	135.0727	1.168	2-Phenylacetamide	0.001	−0.283
1.965	182.0816	1.168	Mannitol	0	−0.328
1.902	164.0516	1.168	Phenylpyruvic acid	0	−0.316
1.869	181.0787	1.168	Tyrosine	0	−0.312
1.607	607.0912	1.168	UDP-N-acetyl-D-galactosamine	0.006	−0.374
1.553	232.114	1.283	4-(Glutamylamino) butanoate	0.006	−1.937
1.443	215.1456	4.848	Pantothenic acid	0.013	0.617
1.372	515.2961	11.163	Taurocholic acid	0.029	1.038
1.861	352.2334	11.514	PGE2	0.001	−0.544
1.545	273.2751	14.728	C16 sphinganine	0.007	−0.427
2.749	287.2907	15.192	C17 sphinganine	0	−2.162
1.71	301.3064	15.54	Sphinganine	0.002	−0.42
1.545	330.341	16.062	Clupanodonic acid	0.007	−0.374
1.493	392.2982	17.603	Deoxycholic acid	0.01	−0.635
1.22	327.2855	17.75	N-palmitoyl alanine	0.048	−0.327
1.293	281.2798	17.751	Oleamide	0.033	−0.342
2.068	337.3424	17.864	Docosenamide	0	−1.448

^∗^Fold change was calculated as the ratio of the mean metabolite levels between two groups. A positive value of fold change indicates a relatively higher concentration of metabolites while a negative value of fold change indicates a relatively lower concentration in infertile males with KYDS as compared to fertile males.

**Table 3:** Identified discriminating metabolites in negative mode.

VIP	m/z	Retention time (min)	Name	t test	Fold change^*^
1.335	446.0562	0.704	CDP-ethanolamine	0.032	−0.61
1.499	136.0298	0.779	Hypoxanthine	0.012	−0.597
1.557	276.0882	0.788	thymidine glycol	0.008	−0.817
1.441	148.0298	0.844	Citramalic acid	0.017	−0.367
1.364	174.009	0.869	Dehydroascorbic acid	0.027	−0.51
1.404	130.0194	0.879	Glutaconic acid	0.022	−0.6
1.556	315.0483	1.134	5′-Phosphoribosyl-N-formylglycinamide	0.008	−0.68
1.39	192.0193	1.142	Citric acid	0.023	−0.363
1.546	112.009	1.143	Furoic acid	0.009	−0.424
1.344	129.0353	1.171	Pyroglutamic acid	0.03	0.675
1.277	426.0053	1.199	APS/ADP/dGDP	0.043	−0.358
1.698	181.0666	1.2	Tyrosine	0.003	−0.476
1.705	130.0194	1.274	Citraconic acid	0.003	−0.655
1.729	174.009	1.275	Aconitic acid	0.002	−0.643
1.298	489.2731	2.654	PA(21:4)	0.038	0.443
1.397	182.0504	4.672	Homovanillic acid	0.022	−0.482
1.458	129.0352	4.923	Pyrroline hydroxycarboxylic acid	0.016	0.597
1.765	545.2636	9.368	PS(20:4)	0.002	−1.924
1.505	370.2288	11.507	6-Keto-PGF1*α*/TXB2	0.012	−0.415
1.516	531.0692	12.557	CDP-4-dehydro-3,6-dideoxy-D-glucose	0.011	−0.428
2.335	337.2285	13.096	PGA1	0	0.817
1.561	335.2129	13.332	PGA2	0.008	1.066
1.267	256.2323	18.876	Palmitic acid	0.045	0.416

^∗^Fold change was calculated as the ratio of the mean metabolite levels between two groups. A positive value of fold change indicates a relatively higher concentration of metabolites while a negative value of fold change indicates a relatively lower concentration in infertile males with KYDS as compared to fertile males.
