# Supplementary material for: Metabolomics Analysis of Seminal Plasma in Infertile Males with Kidney-Yang Deficiency: A Preliminary Study
Source: Evid Based Complement Alternat Med. 2015 Apr 7;2015:892930. doi: 10.1155/2015/892930 (PMC4405216; doi:10.1155/2015/892930)
Supplement: Supplementary file 1 — Additional file 1 was table of gradient elution program of mobile phase. Additional file 2 were typical total ion chromatograms of seminal plasma samples. (a) showed a typical total ion chromatogram from sample of fertile males in positive ion mode, (b) showed a sample of fertile males in negative ion mode, (c) showed a sample of infertile males with KYDS in positive ion mode, and (d) showed a sample of infertile males with KYDS in negative ion mode. [file 892930.f1.pdf]

Additional files

Additional file 1 Gradient elution program of mobile phase

Time (min)	A% (0.1% formic acid in water)	B % (0.1% formic acid in acetonitrile)
0	95	5
2	95	5
17	5	95
19	5	95

Additional file 2 Typical total ion chromatograms of seminal plasma samples

(a) Sample of fertile males in positive ion mode; (b) sample of fertile males in negative ion mode; (c) sample of infertile males with KYDS in positive ion mode; (d) sample of infertile males with KYDS in negative ion mode.

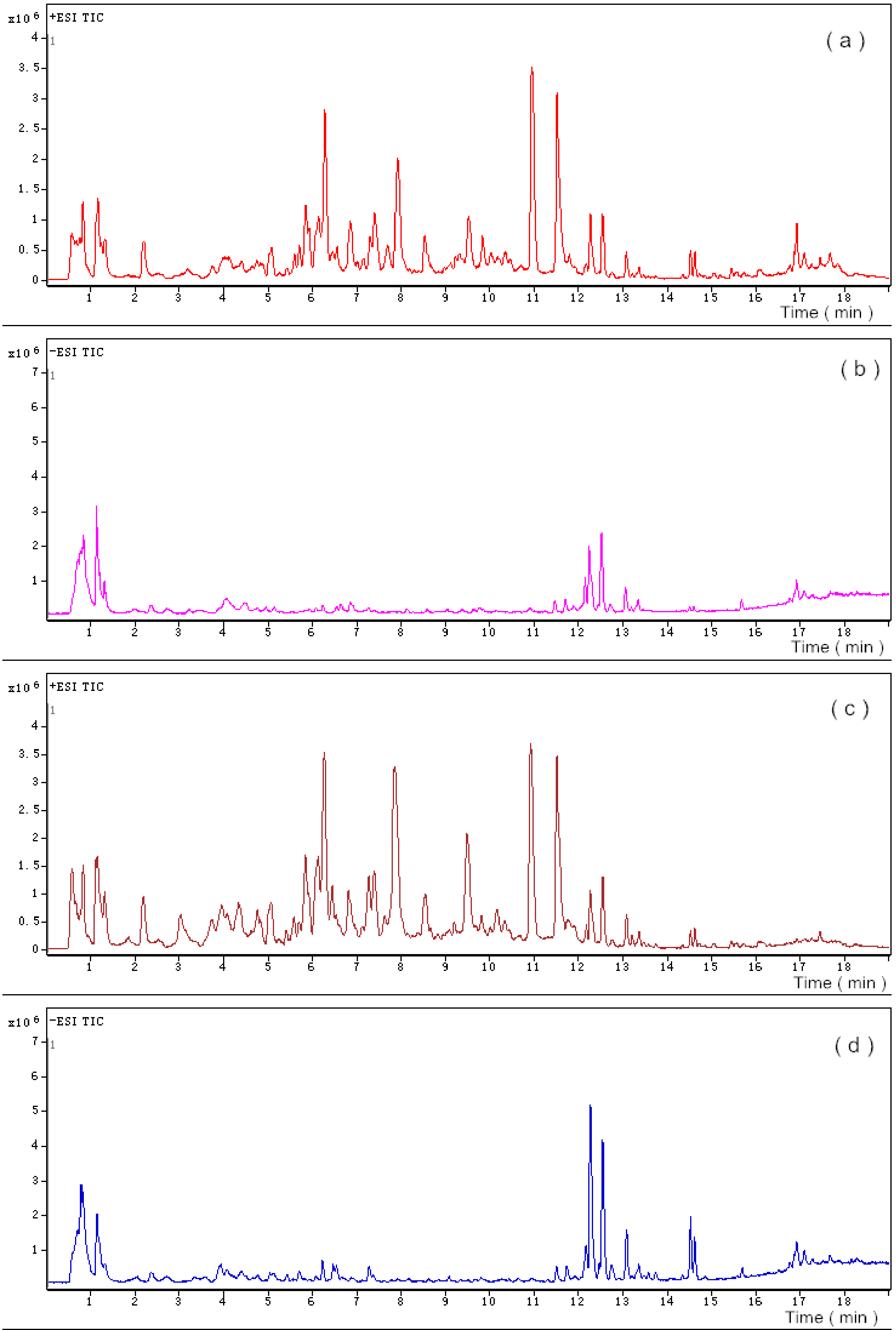
x10 6
+ESI TIC
(a)
Time (min)
x10 6
-ESI TIC
(b)
Time (min)
x10 6
+ESI TIC
(c)
Time (min)
x10 6
-ESI TIC
(d)
Time (min)
